# An Efficient Synthesis and Photoelectric Properties of Green Carbon Quantum Dots with High Fluorescent Quantum Yield

**DOI:** 10.3390/nano10010082

**Published:** 2020-01-01

**Authors:** Jingxia Zheng, Yanting Xie, Yingying Wei, Yongzhen Yang, Xuguang Liu, Yongkang Chen, Bingshe Xu

**Affiliations:** 1 Key Laboratory of Interface Science and Engineering in Advanced Materials, Taiyuan University of Technology, Ministry of Education, Taiyuan 030024, China; 2 Institute for New Carbon Materials, Taiyuan University of Technology, Taiyuan 030024, China; 3 School of Engineering and Computer Science, University of Hertfordshire, Hertfordshire AL10 9AB, UK

**Keywords:** high product yield, green carbon quantum dots, high fluorescent quantum yield, white LEDs

## Abstract

To greatly improve the production quality and efficiency of carbon quantum dots (CQDs), and provide a new approach for the large-scale production of high-quality CQDs, green carbon quantum dots (g-CQDs) with high product yield (PY) and high fluorescent quantum yield (QY) were synthesized by an efficient one-step solvothermal method with 2,7-dihydroxynaphthalene as the carbon source and ethylenediamine as the nitrogen dopant in this study. The PY and QY of g-CQDs were optimised by adjusting reaction parameters such as an amount of added ethylenediamine, reaction temperature, and reaction duration. The results showed that the maximum PY and QY values of g-CQDs were achieved, which were 70.90% and 62.98%, respectively when the amount of added ethylenediamine, reaction temperature, and reaction duration were 4 mL, 180 °C, and 12 h, respectively. With the optimised QY value of g-CQDs, white light emitting diodes (white LEDs) were prepared by combining g-CQDs and blue chip. The colour rendering index of white LEDs reached 87, and the correlated colour temperature was 2520 K, which belongs to the warm white light area and is suitable for indoor lighting. These results indicate that g-CQDs have potential and wide application prospects in the field of white LEDs.

## 1. Introduction

As fluorescent carbon nanomaterials with a size less than 10 nm, carbon quantum dots (CQDs) have good water solubility, excellent chemical inertness, unique resistance to light bleaching, low toxicity, and good biocompatibility, etc. Due to their unique optical properties of tunable photoluminescence and good fluorescent stability, CQDs have been widely used in white light emitting diodes (white LEDs) [1,2,3,4]. In particular, published experimental results have shown that CQDs have great application potential in white LEDs as phosphors [5,6,7].

In the current application of white LEDs, most of the CQDs emitting blue light under ultraviolet light are correlated to colour temperature (CCT) as the basic performance index of white LEDs, and can also change the exhibited colour of the object. The CCT of white LEDs prepared with blue CQDs as phosphor generally exceeds 6000 K, which belongs to cold white light and is suitable for both outdoor and office lighting. However, the introduction of green CQDs (g-CQDs) can significantly reduce the CCT of white LEDs, which can meet the requirements of indoor lighting (CCT < 5000 K) and thus broaden the application of CQDs in white LEDs [8]. Furthermore, with the rapid development of white LEDs, the demand of white LEDs for g-CQDs keeps increasing, where a high product yield (PY) of CQDs is required [6,8,9]. At the same time, the greater demand in brightness and efficiency of white LEDs needs the higher quantum yield (QY) of CQDs, therefore an efficient method to synthesize g-CQDs with both high PY (>50%) and high QY (>50%) needs to be explored.

Many researchers have recently synthesized g-CQDs with a lower value of QY of g-CQDs, which is generally lower than 50% [3,5,8,9,10,11,12,13,14]. For example, Du et al. [8] used pyrogallic acid as a carbon source, and the QY value of g-CQDs obtained by the one-step solvothermal method was 16.8%. Qu et al. [9] adopted the mass ratio of 1:2 for citric acid and urea and used ethanol as the solvent. Their QY value of g-CQDs obtained by the microwave method was 36%. Zhang et al. [10] prepared g-CQDs with a QY value of 44.8% by treating an L-valine aqueous solution with concentrated phosphoric acid at 90 °C. Not much attention has been paid to the PY of g-CQDs, and their PY and QY values are generally low. However, Cui et al. [11] did synthesize g-CQDs with good PY and QY values of 34% and 16.5%, respectively, by the two-step hydrothermal method with the employment of citrate amine as the main raw material. Therefore, there is an urgent to explore novel methods of synthesizing g-CQDs with both high PY (>50%) and high QY (>50%).

Herein, in this work, an efficient one-step solvothermal approach was developed to controllably prepare g-CQDs with high PY (>50%) and high QY (>50%) using 2,7-dihydroxylnaphthalene as the single precursor and ethylenediamine as the nitrogen dopant. The benzene ring and large conjugated structure of 2,7-dihydroxylnaphthalene are expected to increase the electronic cloud density, which is conducive to synthesize CQDs with low band gap and thus realize long-wavelength emissions [15,16,17]. Additionally, 2,7-dihydroxylnaphthalene has abundant carbon–carbon double atoms, which is beneficial to the high PY of CQDs [18]. In addition, ethylenediamine as the nitrogen dopant is expected to improve the QY of CQDs [19,20,21]. Furthermore, the effect of an additive amount of ethylenediamine, reaction temperature, and reaction duration on the PY and QY of g-GQDs was investigated and optimised. Finally, benefiting from high PY and high QY, white LEDs were fabricated with g-CQDs as phosphors combined with a blue chip (λ_em_ = 460 nm).

## 2. Materials and Methods 

### 2.1. Materials

2,7-dihydroxylnaphthalene, ethylenediamine (EDA), hydrogen peroxide (H_2_O_2_, 30 wt%), and anhydrous ethanol were all provided by Tianjin Guangfu Chemical Reagent Co. Ltd. (Tianjin, China) The materials and reagents used were analytically pure in grade.

### 2.2. Synthesis of Green Carbon Quantum Dots (g-CQDs)

**Figure 1:**
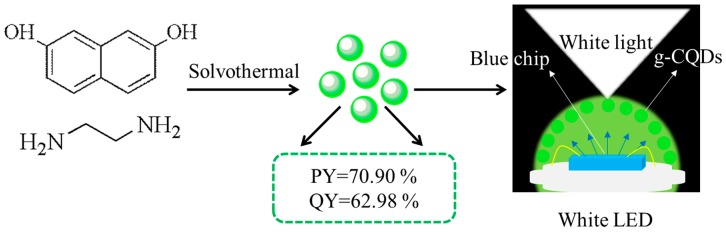
Schematic diagram of the synthesis of green carbon quantum dots (g-CQDs) and their application in white light emitting diodes (white).

2,7-dihydroxylnaphthalene (0.4 g), EDA (0, 2, 4 or 8 mL), H_2_O_2_ (2 mL), and anhydrous ethanol (40 mL) were added into a 100 mL Teflon stainless steel autoclave in turn, which was heated to the temperatures of 160, 180, or 200 °C, respectively, for a period of time (10, 12, or 14 h). After the reaction, it was cooled to the room temperature, and the colour of the solution appeared from colourless to dark wine red. It was then centrifuged with ethyl acetate for 5 min at the rotation speed of 8000 rpm, and repeated three times. Finally, it was dried at 50 °C for 6 h in an oven to obtain g-CQDs powder for later characterisation. The specific schematic diagram is shown in Figure 1. In order to obtain g-CQDs with both high PY (>50%) and high QY (>50%), the effects of a targeted amount of ethylenediamine (V_(EDA)_), reaction temperature (T), and reaction duration (D_r_) were investigated.

### 2.3. Preparation of White Light Emitting Diodes (White LEDs)

The blue light LED chip was purchased from Shenzhen Guanghuashi Technology Co. Ltd. (Shenzhen, China), and its emission wavelength centre is 460 nm, which is fixed at the bottom of the LED base. Two threads on the LED were used to connect the external power supply. Then, the g-CQD solution was gently dropped onto the inner wall of the optical lens, which was then dried in the oven at 80 °C for three hours. Finally, the optical lens was firmly fixed on the top of the LED chip to realize the fabrication of white LEDs.

### 2.4. Characterisation

Transmission images (TEM) and high-resolution transmission image (HRTEM) of the g-CQDs were obtained by a JEOL JEM-2010 microscope. The powder sample of g-CQDs was first dispersed in ethanol ultrasonically and then dripped onto ultra-thin carbon films. The X-ray diffraction (XRD) pattern of the g-CQDs was recorded on a Rigaku-D/MAX 2500 diffractometer equipped with graphite monochromatized Cu Kα (λ = 1.54 Å) radiation at a scanning speed of 4°/min in the 2θ range from 10° to 90°. The sample for XRD was pressed on the glass substrate. The Fourier transform infrared (FTIR) spectra of the g-CQDs using KBr pellets were measured by a Bruker Tensor 27 spectrometer. X-ray photoelectron spectroscopy (XPS) measurements were performed on a Kratos AXIS ULTRA DLD X-ray photoelectron spectrometer with a single X-ray source AlK excitation (1486.6 eV). An ELEMENTAR vario EL cube was used for elemental analysis (EA). The UV–Vis absorption spectra were determined by a Hitachi U3900 UV–Vis spectrophotometer. The excitation and emission spectra of the g-CQD solution were carried out using the Horiba Fluoromax-4 fluorescence spectrometer with an Xe lamp as the excitation source. An FLS 980 transient fluorescence spectrometer was employed to measure the fluorescent decay curve and calculate the fluorescence lifetime of g-CQDs. Spectra Scan PR 655 was applied to analyse the spectra, Commission Internationale de l’Eclairage (CIE) coordinates, colour rendering index (CRI) and correlated colour temperature (CCT) of the white LEDs.

### 2.5. Calculation of Quantum Yield (QY)

The QY of the g-CQDs was measured by a relative method. Rhodamine B aqueous solution was selected as the standard solution to determine the relative QY of the g-CQD solution. The absorbance values of all solutions were measured at the excitation wavelength of 460 nm and kept below 0.1 to minimize the reabsorption effect. The integral emission intensity was the area under the 480–700 nm emission curves. Draw the linear fitting line of integral emission intensity as the ordinate and absorbance as the abscissa and obtain the slope of the linear fitting line. Finally, the QY value was calculated according to Equation (1):

Q = Q_st_(K/K_st_)(η/η_st_)^2^ (1)

where Q is the QY; K is the slope of the linear fitting line; and η is the index of refraction of the solvent. In this experiment, η = 1.36, η_st_ = 1.33. The subscript “st” refers to the Rhodamine B standard aqueous solution.

### 2.6. Calculation of Product Yield (PY)

The g-CQD solid was obtained after rotary evaporation and centrifugation, and then was weighed on a tray balance. Finally, the PY value was calculated according to Equation (2):

PY = (m_CQDs_/m_c_) × 100% (2)

where m_CQDs_ refers to the mass of the g-CQD solid, and m_C_ refers to the mass of 2,7-dihydroxylnaphthalene.

## 3. Results and Discussion

### 3.1. Influence of Reaction Conditions on PY and QY

In the process of synthesizing g-CQDs, 2,7-dihydroxynaphthalene, EDA, and H_2_O_2_ were added in turn. There was no obvious reaction observed after 2,7-dihydroxylnaphthalene and EDA was added. With the introduction of H_2_O_2_ into the solution, a violent reaction occurred and a large amount of heat was released while bubbling. The solution turned dark wine red when anhydrous ethanol was added. This indicates that after the addition of H_2_O_2_, the EDA has catalysed the decomposition of H_2_O_2_ to release a lot of heat, and at the same time, 2,7-dihydroxylnaphthalene and EDA have undergone a Schiff-base condensation reaction and rapid carbonisation [22], and then g-CQDs have been generated through a solvothermal reaction.

Based on the observations, it appears that the amount of ethylenediamine (V_(EDA)_), reaction temperature (T), and reaction duration (D_r_) are the three major factors affecting both the PY and QY of the g-CQDs. To obtain g-GQDs with high PY and QY (both > 50%), the effect of V_(EDA)_ was investigated. As shown in Table 1, with a gradual increase of the V_(EDA)_, both the PY and QY values of g-CQDs first increased and then decreased. As the formation process of CQDs includes oxidation, amination, and carbonation [22], a change in the PY value is mainly related to the carbonisation process of g-CQDs [23]. As the V_(EDA)_ was increased from 0 to 8 mL, the amination process of g-CQDs was sufficient at the beginning and then excessive. This led to a carbonisation process of g-CQDs gradually reaching a complete and then excessive carbonisation, resulting in the production of a large number of by-products. Consequently, the PY value of g-CQDs first increased from 0.60% (0) to 88.58% (2 mL), and then decreased to 51.60% (8 mL). The change in the QY value of the g-CQDs is directly related to the amount of nitrogen doping. It was observed that with a gradual increase of V_(EDA)_, the amination process of g-CQDs was first full and then excessive, which meant that the nitrogen content of the g-CQDs gradually was increased, and with a gradual increase in nitrogen content, the QY of the g-CQDs first was increased from 5.29% (0) to 62.98% (4 mL) and then decreased to 36.22% (8 mL), which agreed well with the results in the literature [10]. In order to obtain g-CQDs with both high PY and high QY at the same time, V_(EDA)_ was analysed. It appears that when V_(EDA)_ was 4 mL, the QY value of the g-CQDs reached the highest (62.98%) and the PY value reached 70.90%. Therefore, it is suggested that the optimal V_(EDA)_ is 4 mL.

Under the optimal V_(EDA)_ of 4 mL, both the PY and QY values of g-CQDs showed a trend of first increasing and then decreasing with an increase of T. It was observed that the PY value was increased from 21.15% to 70.90% when T was increased from 160 °C to 180 °C and the degree of carbonation was gradually enhanced. When T continued to rise to 200 °C, the PY value decreased to 50.50%. The possible reason for this was that in the range of 160–180 °C, as the carbonation degree was gradually strengthened, PY was gradually increased, and at 180 °C, the carbonation degree was the most appropriate, so that PY reached the maximum. As T continued to rise, the g-CQDs continued to grow along with the generation of a large number of carbon particles and other by-products, resulting in excessive carbonation so that the value of PY declined significantly [21,23]. In contrast, at 160 °C, the QY value was low and 15.07% because the QY was mainly derived from a surface state and the carbonation reaction may be incomplete at this temperature. With T increasing from 160 °C to 180 °C, the carbonation reaction was gradually entirely achieved and the carbon core was gradually formed. At this time, the QY was derived from the synergism of the surface state and carbon core state, and QY was obviously increased and reached 62.98%. With a further increase of T to 200 °C the carbonation reaction was further carried out and the carbon core grew gradually. At this moment, the QY mainly came from the carbon core state, so the QY decreased to 43.05% [24]. Therefore, 180 °C was chosen as the optimal T.

In the meantime, the influence of D_r_ on the PY and QY of the g-GQDs was studied at the optimal T of 180 °C and V_(EDA)_ of 4 mL. It was observed that when D_r_ was increased from 10 h to 14 h, both the PY and QY showed a similar trend of first increasing and then decreasing. When the D_r_ was increased from 10 h to 12 h, the carbon core of the g-CQDs kept growing [21] and a carbonation degree was gradually enhanced, so the PY value was increased from 34.90% to 70.90%. When D_r_ was increased to 14 h, the PY value decreased to 37.10%. This indicates that when the D_r_ was 12 h, the PY value was the highest and the carbonation degree was the most appropriate. Long-time continuous heating might lead to excessive carbonation, causing a dramatic decline in the PY value [23,25]. For the QY, when D_r_ was 10 h, the carbonation reaction was incomplete, and the QY mainly came from the surface state, accompanied by a low QY of 25.42%. With the further extension of D_r_, the carbonation reaction was gradually completed and the carbon core was gradually formed. Under this stage, the QY came from the synergetic effects of the surface state and the carbon core state, hence QY was increased obviously to 62.98% [21,24]. As the reaction duration was prolonged, the carbonisation reaction further proceeded and the carbon core grew gradually. The surface state gradually weakened and the new carbon core state was gradually strengthened at this stage, leading to the decrease of QY to 24.02% [24]. Hence, with a comprehensive consideration of product property and experimental cost, the g-CQDs with a maximum PY value of 70.90% and QY of 62.98% with the optimal D_r_ of 12 h were selected for further investigation of their structure, optical property, and application.

Table 2 exhibits a comparison of the PY and QY values of the g-CQDs synthesised with different materials and methods. As shown in Table 1, the highest QY value of the g-CQDs in this study was 62.98%, which was slightly lower than that obtained by Yuan et al. [12] (80%). Yuan et al. used 3,4,9,10-tetranitroperylene as the raw material, added sodium hydroxide, and obtained g-CQDs by the solvothermal method. The difference in QY is caused by the difference in the nature of the material itself. The 3,4,9,10-tetranitroperylene was rich in nitrogen elements, which was beneficial to its high QY. In terms of the formation mechanism, its high QY (80%) mainly comes from two aspects. On one hand, the high QY of their g-CQDs was due to the alkaline environment (NaOH). The control-experiment showed that QY was 29% with no sodium hydroxide added, which was significantly lower than its maximum QY value of 81%, indicating that the high QY value was closely related to their alkaline environment. On the other hand, the high QY value of their g-CQDs was derived from hydroxyl functionalization. Hydroxyl is a powerful electron donor, containing heteroatoms with unbonded p electrons and can result in the increase of the electron cloud mobility in a conjugated system, which enhances the p–π conjugation between the hydroxyl and the CQDs. As a consequence, the g-CQDs prepared by Yuan et al. [12] had a high QY value. 

As shown in Table 1, the highest PY value of the g-CQDs was 88.58%, and is relatively high at present. This is because 2,7-dihydroxylnaphthalene has many carbon atoms, which contributes to the high PY value. Carbon is conducive to the structural stability of CQDs, so the higher the carbon content, the higher the PY value of the CQDs. It can also be seen in Table 1 that the QY value of the g-CQDs prepared was significantly higher than those of most of the reported g-CQDs, which is mainly because of the addition of EDA [26,27,28,29,30]. As a surface dopant, EDA introduces a new surface state. The fluorescence phenomenon of g-CQDs passivated by EDA is due to the radiation recombination of electrons and holes trapped on the surfaces of g-CQDs [26]. The high QY of g-CQDs may be the result of the interaction between the captured holes on the surfaces of the g-CQDs and the passivation of g-CQDs [27] because nitrogen-containing functional groups are excellent colour cooperators. Correlational studies [29] show that nitrogen-containing groups or nitrogen content play an important role in high QY and it may be that doped nitrogen can passivate the surface active sites by stabilising excitons of g-CQDs, which can greatly improve the fluorescence properties [27,30]. Finally, 2,7-dihydroxylnaphthalene is rich in hydroxyl, which may contribute to the high QY values of g-CQDs [12].

### 3.2. Morphology and Structures

**Figure 2:**
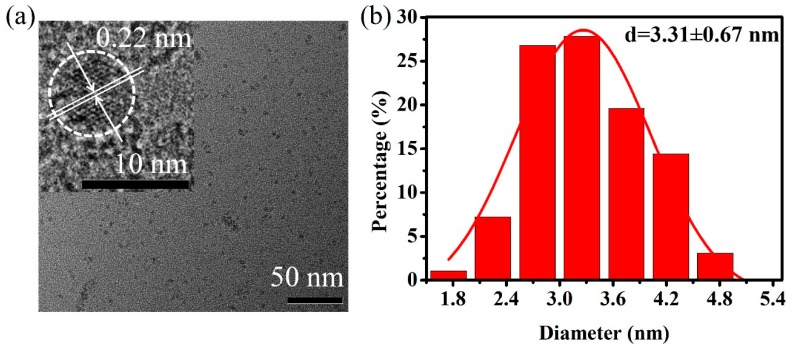
(**a**) Transmission electron microscopy (TEM) image and (**b**) size distribution of g-CQDs, inset of (a) is the high resolution TEM image. (V_(EDA)_ = 4 mL, T = 180 °C, D_r_ = 12 h).

**Figure 3:**
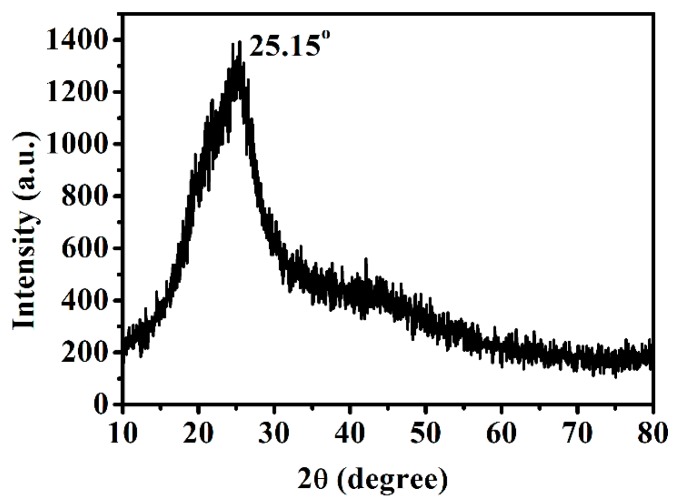
X-ray diffraction of g-CQDs (V_(EDA)_ = 4 mL, T = 180 °C, D_r_ = 12 h).

The morphology structures of g-CQDs were analysed using transmission electron microscopy (TEM). As shown in Figure 2a, g-CQDs are spherical and have uniform dispersion with no obvious aggregation phenomenon. Figure 2b shows that g-CQDs have a narrow size distribution and the average size is 3.31 nm. It can be seen from a high resolution TEM image of the inset in Figure 2a that g-CQDs have an obvious lattice fringe whose spacing is 0.22 nm and close to the graphite (100) crystal plane, which indicates that the g-CQDs have a degree of crystallinity. Meanwhile, according to the X-ray diffraction (XRD) pattern (Figure 3), g-CQDs have a narrow diffraction peak at 2θ = 25.15°, also illustrating the crystallinity structures of g-CQDs.

**Figure 4:**
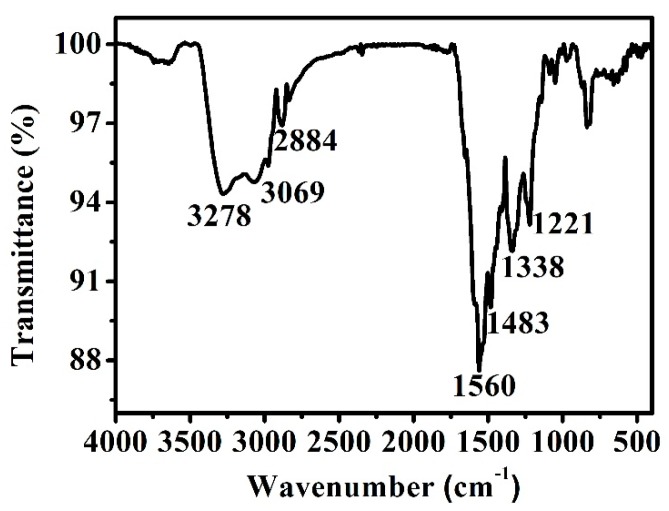
Fourier transform infrared (FTIR) spectrum of the g-CQDs (V_(EDA)_ = 4 mL, T = 180 °C, D_r_ = 12 h).

In order to better analyse the surface state of g-CQDs, the structures of g-CQDs were characterised by Fourier transform infrared (FTIR) spectroscopy. Figure 4 shows that the absorption peak at 3278 cm^−1^ can be attributed to the stretching vibration of O–H and N–H, which proves that g-CQDs have a certain degree of nitrogen doping and promoted the improvement of QY. The absorption peak at 3069 cm^−1^ was attributed to the stretching vibration of O–H, while the absorption peak at 2884 cm^−1^ was attributed to the vibration of C–H and O–H. The 1560 cm^−1^ peak was attributed to the vibration of C=N and C=C. The absorption peak at 1483 cm^−1^ was attributed to the vibration of C=N, C=C, and C–OH. The absorption peak at 1338 cm^−1^ was attributed to the vibration of C–N, while the absorption peak at 1221 cm^−1^ was attributed to the vibration of C–O–C, =C–H and C–OH. These indicate that g-CQDs have abundant functional groups such as the hydroxyl group and amino group. 

**Figure 5:**
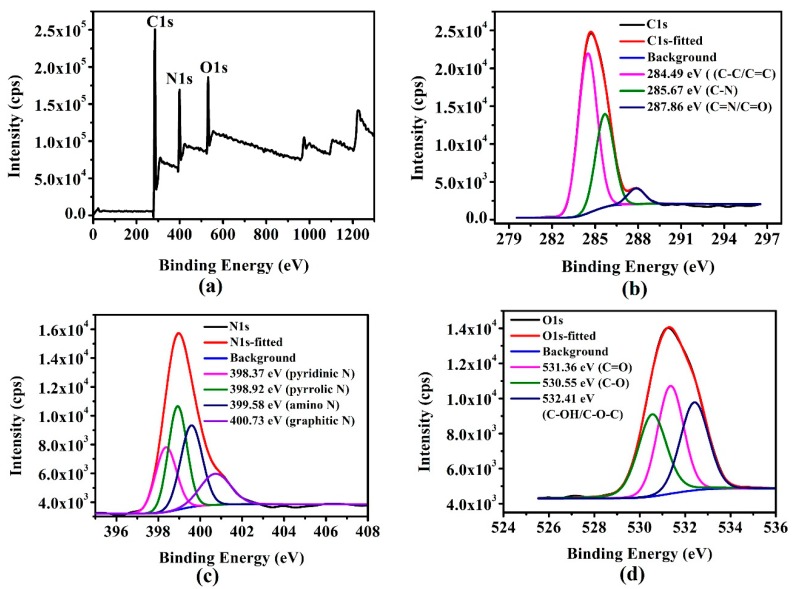
(**a**) X-ray photoelectron spectroscopy (XPS) spectrum of the g-CQDs, and (**b**–**d**) are the corresponding extended peaks of C1s, N1s and O1s, respectively (V_(EDA)_ = 4 mL, T = 180 °C, D_r_ = 12 h).

To further characterise the chemical bonds, the elemental composition and functional group properties of the surfaces of the g-CQDs, X-ray photoelectron spectroscopy (XPS) technology was applied. A full-scan XPS spectrum of the g-CQDs is shown in Figure 5a. Strong C1s and O1s peaks in Figure 5a indicate that the g-CQDs are mainly composed of carbon elements and oxygen elements, while the N1s peak indicates that EDA is involved in the formation of g-CQDs. Elemental analysis (EA) was used to analyse the element types and the percentage content of g-CQDs. As shown in Table 3, the EA results of the g-CQDs were consistent with the full-scan XPS spectrum of g-CQDs. The peak at 284.49 eV of the C1s spectrum in Figure 5b was attributed to C–C/C=C. Two peaks centred at 285.67 and 287.86 eV corresponded to C–N and C=N, respectively. The introduction of the N elements comes from EDA doping, which proves that 2,7-dihydroxynaphthalene reacted with the amination process. The N1s spectrum in Figure 5c also illustrates this result: the introduction of nitrogen atoms increases the density of the electron cloud on the surfaces of the g-CQDs and promotes long wavelength emissions [29]. In addition, nitrogen doping promotes the improvement of QY [27,28,30,31]. Figure 5d illustrates the O1s XPS spectrum of g-GQDs, which was deconvoluted into three peaks at 531.36, 530.55, and 532.41 eV ascribed to C=O, C–O, and C–OH/C-O-C, respectively. This indicates that 2,7-dihydroxylnaphthalene has been oxidised to further form g-CQDs.

### 3.3. Optical Properties

**Figure 6:**
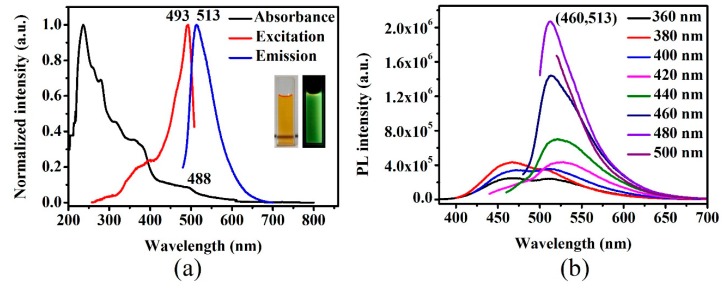
(**a**) UV–Vis absorption spectrum and excitation spectrum of g-CQD solution; (**b**) fluorescence spectra of g-CQD solution at different excitation wavelengths (V_(EDA)_ = 4 mL, T = 180 °C, D_r_ = 12 h).

The optical properties of g-CQDs under the optimal reaction conditions (V_(EDA)_ = 4 mL, T = 180 °C, D_r_ = 12 h (at this time, the amount of added 2,7-dihydroxylnaphthalene was 0.4 g)) were characterised. The UV–Vis absorption spectrum of the g-CQD solution as shown in Figure 6 indicates that g-CQDs have an obvious absorption peak at 488 nm, which can be attributed to the n–π* transition of the C=O bond. In addition, the excitation spectrum of g-CQDs also has an apparent excitation peak at 493 nm. The excitation spectrum of g-CQDs corresponds to their absorption spectrum. The fluorescence emission spectra of g-CQDs show that the emission peak of the g-CQDs shifted significantly as the excitation wavelength increased from 360 nm to 500 nm, demonstrating that g-CQDs have the property of excitation wavelength dependence, which may be related to the uneven surface state of g-CQDs or the polydispersity of g-CQDs, considering Figure 2b. When the excitation wavelength was 460 nm, the emission peak of g-CQDs was 513 nm, showing green fluorescence.

**Figure 7:**
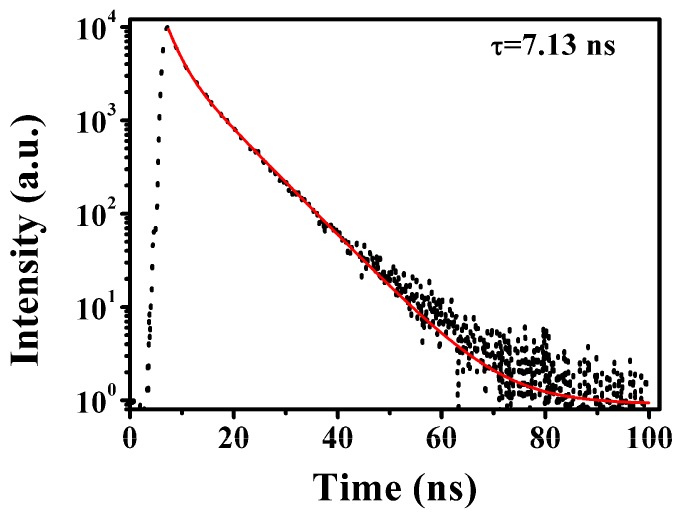
Fluorescence decay curve of g-CQDs (V_(EDA)_ = 4 mL, T = 180 °C, D_r_ = 12 h).

In order to better combine g-CQDs with white LEDs, a multi-dimensional time-dependent single photon count (TCSPC) method was used to evaluate the fluorescence decay behaviour of g-CQDs. As shown in Figure 7, the fluorescence decay curve of g-CQDs was fitted according to the double-exponential model of Equation (3):

R(t) = α_1_exp(−t/τ_1_)α_2_exp(−t/τ_2_) (3)

The average lifetime was calculated according to Equation (4): where α_1_ and α_2_ are the percentages of the decay lifetime of τ_1_ and τ_2_, respectively.

τ = (α_1_τ_1_^2^ + α_2_τ_2_^2^)/(α_1_τ_1_ + α_2_τ_2_) (4)

It is reported that the fluorescence mechanism of CQDs is closely related to its surface state and carbon core state [31]. The two radiation lifetimes τ_1_ and τ_2_ can be ascribed to the internal recombination of carbon core state and surface state, respectively [32,33]. The fluorescence decay curve of g-CQDs in Figure 7 and the fitting parameters in Table 4 indicate that g-CQDs present a double exponential decay with an average lifetime of 7.13 ns. The fluorescence lifetime of g-CQDs consists of two parts. Among them, the proportion of τ_2_ (long-life components) is 72%, which plays a leading role in the radiation lifetime, indicating that the fluorescence decay kinetics of g-CQDs is mainly caused by their surface state, and their green fluorescence may mainly come from their surface rich functional groups.

**Figure 8:**
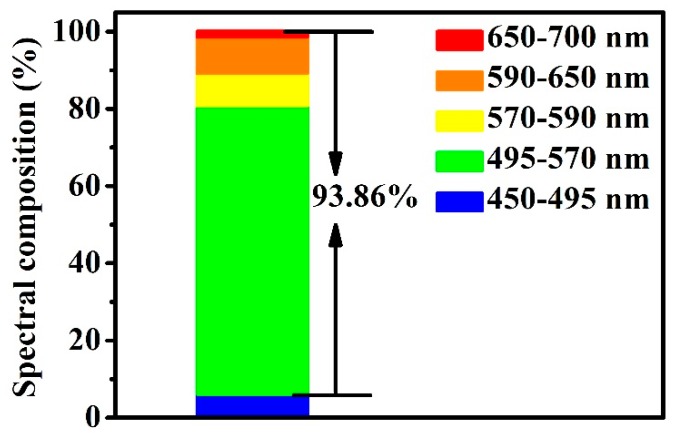
The red–green–blue spectral composition of g-CQDs (V_(EDA)_ = 4 mL, T = 180 °C, D_r_ = 12 h).

As shown in Figure 8, the red–green–blue spectral composition (RGB) of the g-CQDs was 93.86%. High RGB is in favour of improving the efficiency of converting blue light into white light, which is conducive to the high colour rendering index of white LEDs [10] as the high RGB can be attributed to the high electron density on the surfaces of the g-CQDs. This will reduce the energy gap of the g-CQD surface state transition, resulting in a red shift in the emission wavelength [34], which will make g-CQDs as phosphor more suitable for white LEDs application.

**Figure 9:**
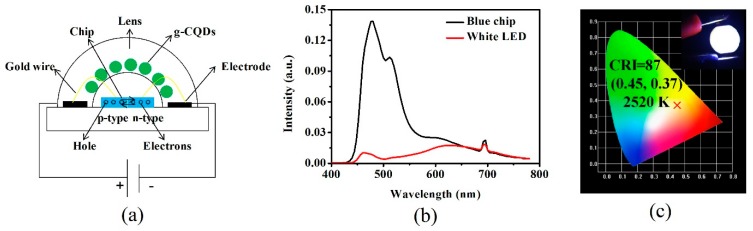
Schematic diagram of (**a**) the structure of white LEDs based on g-CQDs (V_(EDA)_ = 4 mL, T = 180 °C, D_r_ = 12 h); (**b**) emission spectrum of the single blue light chip and white LEDs; (**c**) CIE chromaticity diagram of white LEDs, inset is the photo of white LEDs.

With their excellent optical performance, g-CQDs were combined with a blue light chip to prepare white LEDs in order to further explore the potential application of g-CQDs in white LEDs. LEDs mainly consisted of a blue chip, gold wire, optical lens, and electrode, as shown in Figure 9a. The blue light chip was the excitation light, and the optical lens coated with g-CQDs was the white light converter. One end of the chip was connected to the cathode of the power source and the other end was connected to the anode. When the current was applied, a p–n junction would be formed, which promoted electron-hole recombination and photon release energy. Subsequently, g-CQDs absorbed the excited light from the blue light chip and re-emitted visible light, and finally converted blue light into white light [6]. The emission spectrum of white LEDs was recorded.

The results show that the emission spectrum of the white LED device covers the entire visible region (400–780 nm) (Figure 9b). The colour coordinates of white LEDs are (0.45, 0.37), the colour rendering index (CRI) is 87, and the CCT is 2520 K, which belongs to the warm white light area and is suitable for indoor lighting (Figure 9c). The warm white LEDs emitting bright warm light with high CRI were comparable to the white LEDs based on other CQDs, semiconductor QDs, and rare-earth phosphors in Table 5 [12,35,36,37,38,39]. However, these articles did not take the PY of CQDs into consideration. In this work, g-CQDs with high PY were synthesized under the premise of ensuring a high QY of g-CQDs. These results manifest the feasibility of the green emission of CQDs as the single phosphor in the fabrication of white LEDs.

## 4. Conclusions

In summary, g-CQDs with both high PY (70.90%) and high QY (62.98%) were synthesised with 2,7-dihydroxylnaphthalene by a one-step solvothermal method. The studied results showed that the additive amount of ethylenediamine, reaction temperature, and reaction duration had an important influence on the PY and QY of g-GQDs and the optimized parameters were 4 mL, 180 °C, and 12 h, respectively. It was also found that abundant carbon atoms are beneficial to the high PY of CQDs, and high nitrogen doping content and rich hydroxyl functional groups contribute to improving the QY of CQDs. The synthesised g-CQDs were spherical with the average particle size of 3.31 nm. The g-CQD solution emits bright green fluorescence under ultraviolet light and has the characteristic of excitation wavelength dependence. Taking advantage of the high QY of g-CQDs, the colour coordinates of the as-prepared white LED devices were (0.45, 0.37), the colour rendering index was 87, and the CCT was 2520 K. The results show that g-CQDs have great application potential in the field of indoor lighting. Furthermore, due to the low cost of raw materials and the simplicity of the synthesis method, this study explored a simple and efficient method for the synthesis of high-quality g-CQDs, which will not only broaden the further application of CQDs, but more importantly, open up a new and efficient method for the large-scale preparation of CQDs in the field of LED lighting.

## Figures and Tables

**Table 1:** Product yield (PY) and quantum yield (QY) values of green carbon quantum dots (g-CQDs) under different reaction conditions.

V_(EDA)_/mL	Reaction Temperature/°C	Reaction Duration/h	PY/%	QY/%
0	180	12	0.60	5.29
2			88.58	41.07
4			70.90	62.98
8			51.60	36.22
4	160	12	21.15	15.07
	180		70.90	62.98
	200		50.50	43.05
4	180	10	34.90	25.42
		12	70.90	62.98
		14	37.10	24.02

**Table 2:** A comparison of the QY and PY of g-CQDs synthesised by different materials and methods.

Year	Experimental Materials	Synthesis Methods	PY/%	QY/%	References
2018	3,4,9,10-tetranitroperylene	solvothermal	-	80	[12]
2018	citric acid, N-(2-aminoethyl)-3- aminopropyltrimethoxysilane (AEATMS)	solvothermal		49	[3]
2018	urea, aniline, ethylenediamine	hydrothermal	-	2.46	[13]
2017	citric acid, N-(β-aminoethyl)-γ-aminopropyl trimethoxysilane (AEAPMS)	solvothermal	-	16.4	[5]
2015	L-valine, H_3_PO_4_ (85%)	solvothermal	-	44.8	[10]
2014	citric acid, urea	solvothermal	-	36	[9]
2014	ammonium citrate, ammonium hydroxide, H_2_O_2_	hydrothermal	34	16.5	[11]
2013	phytic acid, ethylenediamine	microwave oven	-	21.65	[14]
2018	2,7-dihydroxynaphthalene, ethylenediamine, H_2_O_2_	solvothermal	70.90	62.98	this article

**Table 3:** Elemental analysis results of the g-CQDs (V_(EDA)_ = 4 mL, T = 180 °C, D_r_ = 12 h).

Sample	N	C	H	O
g-CQDs/%	20.31	55.15	5.44	19.10

**Table 4:** Double-exponential fitting parameters of the g-CQD solution (V_(EDA)_ =4 mL, T = 180 °C, D_r_ = 12 h).

Sample	α_1_	τ_1_ (ns)	α_2_	τ_2_ (ns)	χ^2^	<τ> (ns)
g-CQDs	0.28	2.18	0.72	7.68	1.28	7.13

**Table 5:** Comparison of LEDs fabricated by the blue chip and different g-CQDs and other phosphors.

Products	PY/%	QY/%	λ_ex_/nm	λ_em_/nm	WLEDs				References
					Phosphor	CIE Coordinates	CRI	CCT	
CDs	-	16.5	400	500	CDs + CaAlSiN_3_:Eu^2+^	(0.382, 0.391)	86.9	3863	[35]
SiCDs	-	-	450	524	SiCDs	(0.3353, 0.5647)	-	-	[36]
G-CDs	-	80	460	508	G-CDs/MTES + R-CDs/APTES	(0.4046, 0.4028)	92.9	3610	[12]
g-CDs	-	14	405	522	g-CDs@MMT composites	(0.46, 0.49)	-	3232	[37]
(CdSe)_x_(ZnS)_1−x_	-	54	365	517	(CdSe)_x_(ZnS)_1−x_ + (CuInS_2_)_x_(ZnS)_1−x_	(0.437, 0.432)	65.5	3220	[38]
Sr_7.95_Si_4_O_12_Cl_8_: 0.05Eu^2+^	-	81	395	500–557	Sr_7.95_Si_4_O_12_Cl_8_:0.05 Eu^2+^ + Ca^2+^/Sr^2+^/Mn^2+^	~	90.3	~	[39]
g-CQDs	70.90	62.98	460	513	g-CQDs	(0.45, 0.37)	87	2520	this article

MTES: methyltriethoxysilane; APTES: 3-triethoxysilylpropylamine; “~” refers to variable.
